# Endoscopic Ultrasound-Guided Locoregional Treatments for Solid Pancreatic Neoplasms

**DOI:** 10.3390/cancers15194718

**Published:** 2023-09-25

**Authors:** Luca Di Gialleonardo, Giulia Tripodi, Gianenrico Rizzatti, Maria Elena Ainora, Cristiano Spada, Alberto Larghi, Antonio Gasbarrini, Maria Assunta Zocco

**Affiliations:** 1 CEMAD Digestive Diseases Center, Fondazione Policlinico Universitario “A. Gemelli” IRCCS, Largo A. Gemelli 8, 00168 Rome, Italy; lucadigialleonardo9292@gmail.com (L.D.G.); ainoramariaelena@gmail.com (M.E.A.); antonio.gasbarrini@unicatt.it (A.G.); 2 Digestive Endoscopy Unit, Fondazione Policlinico Universitario “A. Gemelli” IRCCS, Largo A. Gemelli 8, 00168 Rome, Italy; giulia.tripodi91@gmail.com (G.T.); gianenrico.rizzatti@gmail.com (G.R.); cristiano.spada@policlinicogemelli.it (C.S.); alberto.larghi@policlinicogemelli.it (A.L.)

**Keywords:** solid pancreatic neoplasms, endoscopic ultrasound-guided local treatment, thermal ablative techniques, non-thermal injection techniques

## Abstract

**Simple Summary:**

Endoscopic ultrasound-guided locoregional treatments actually represent a leading role in the majority of solid pancreatic neoplasms, with the aim to combine with optimal management of clinical symptoms for a better quality of life. Recently, several endoscopic ultrasound-guided locoregional treatment techniques for solid pancreatic neoplasms (especially thermal ablative techniques and non-thermal injection techniques) have been developed. The focus of this review is to update evidence about the efficacy and safety of endoscopic ultrasound-guided locoregional treatments in solid pancreatic neoplasms.

**Abstract:**

Solid pancreatic neoplasms are one of the most diagnosed gastrointestinal malignancies thanks to the current and progressive advances in radiologic methods. Endoscopic ultrasound-guided techniques have over time gained a prominent role in the differential diagnosis and characterization of these pancreatic lesions, including pancreatic cancer, neuroendocrine tumors, and metastases. Recently, several endoscopic ultrasound-guided locoregional treatment techniques, which are divided into thermal ablative techniques and non-thermal injection techniques, have been developed and applied in different settings for the treatment of solid pancreatic neoplasms. The most common ablative techniques are radiofrequency, microwave, laser, photodynamic therapy and hybrid techniques such as hybrid cryothermal ablation. The most common injection techniques are ethanol injection, immunotherapy and brachytherapy. In this review, we update evidence about the efficacy and safety of endoscopic ultrasound-guided locoregional treatments for solid pancreatic neoplasms.

## 1. Introduction

In recent years, Endoscopic Ultrasound (EUS) has rapidly shifted from a purely diagnostic procedure to a wide range of interventional procedures. The constant pursuit of less invasive approaches has been the main driver for EUS scope evolution and the development of dedicated accessories.

The introduction of lumen-apposing metal stents (LAMSs) allowed to perform procedures such as pancreatic collection, biliary and gallbladder drainages with a single device, in fewer steps and also creating a stable and wide connection [1].

Furthermore, the ability of LAMS to create stable anastomosis has made it possible to perform EUS-guided gastrojejunal anastomoses similarly to those performed in conventional surgery but with a minimally invasive approach [2].

Finally, the capability of EUS reaching deep organs prompted the development of dedicated devices to perform various locoregional treatments, in particular for solid pancreatic neoplasms [3].

This review will focus on locoregional ablative techniques under EUS guidance, which can be broadly divided into thermal ablative and non-thermal injection techniques.

A PubMed/Medline database research was performed before August 2023 to identify studies concerning EUS-guided locoregional treatments for solid pancreatic neoplasms. The selected keywords were EUS or endoscopic ultrasound and pancreas or pancreatic associated with any of the following: ablative, ablation, radiofrequency, injection, neuroendocrine tumor or neoplasm, insulinoma, carcinoma or adenocarcinoma, tumor or neoplasm, brachytherapy, implantation, treatment or therapy, intratumoral. Only articles in the English language were included.

## 2. Thermal Ablative Procedures

Thermal ablative procedures include radiofrequency (RF), microwave (MW), laser (LA), photodynamic therapy (PDT), and hybrid techniques such as hybrid cryothermal ablation.

All these techniques are based on the generation of heat that induces irreversible cellular damage, cellular apoptosis and coagulative necrosis [4]. In addition, activation of the immune system due to the release of tumoral antigens also seems to play a role in the therapeutic ablative effect, not only on the target lesion but potentially also on other non-targeted lesions, the so-called abscopal effect [5].

Studies about thermal ablative procedures are summarized in Table 1.

### 2.1. Radiofrequency Ablation

Radiofrequency ablation (EUS-RFA) is the most established and studied technique, and it has been used for the treatment of several abdominal neoplasms, mainly for hepatocellular carcinoma or liver metastasis [6] as well as for pancreatic cancer [7].

EUS-RFA is now performed using dedicated devices inserted through the working channel of the scope. The most used and currently only available device is the Taewoong system (Taewoong, Gimpo-si, Gyeonggi-do, South Korea), consisting of a 19G electrode needle (EUSRA) with an exposed active tip ranging from 5 to 15 mm, coupled with a dedicated radiofrequency generator (VIVA Combo generator, Starmed) and an external cooling system that circulates chilled saline solution through the needle, permitting ablation of large tissue volumes and avoiding tissue charring.

The Habib (EndoHPB) EUS-RFA probe, consisting of a monopolar 1 Fr (0.33 mm) catheter than can be easily inserted through a regular 22G FNA needle, has also been described in the literature [8,9]. Currently, however, this device is unavailable as it was retracted from the market for further improvements.

EUS-RFA has been performed mostly for pancreatic neuroendocrine neoplasms (PNENs) but also for pancreatic adenocarcinoma (PDAC), pancreatic metastases from distant primitive tumors and for selected pancreatic cystic lesions (PCLs) [10,11].

### 2.2. Radiofrequency Ablation in Pancreatic Neuroendocrine Neoplasms

PNENS are further divided into functional (F-) if a clinical syndrome related to the hormonal hypersecretion by the tumor is present, or non-functional (NF-) if the clinical syndrome is absent.

Insulinomas are the most frequent F-pNENs and are considered the ideal candidate for EUS-RFA, as in most cases they have a low malignant potential, while an aggressive behavior is present in less than 10% of cases [12]. As a consequence, there is no strict requirement for a complete tissue ablation but only enough for cessation of the clinical hormonal syndrome.

NF-pNENs, on the other hand, have variable malignant potential, and the ideal patient to be a candidate for a locoregional treatment is not well established. Factors such as Ki67 grade, presence of symptoms, calcifications and in particular lesion size that contribute to the malignant potential of PNENs should be taken into account before proposing ablative procedures in these patients [13].

In patients with NF-PNENs, a complete ablation is thus required as the treatment objective is to limit tumor progression, as well as lymph nodes and distal involvement.

The experience of EUS-RFA in patients with PNENs is mostly limited to case reports, small case series and retrospective studies with limited numbers of patients.

Regarding F-PNENS, Oleinikov and colleagues enrolled 7 patients with insulinomas treated with EUS [14]. All patients were successfully treated without significant side effects, obtaining complete regression of the hypersecretion syndrome within 24 h from the procedure. Limitations of this study include a short clinical period (mean follow-up of 8.7 ± 4.6 months) and the lack of radiological FU.

Furnica et al. [15] treated 4 F-pNEN patients with EUS-RFA technique. The treatment was effective in all patients (100%). One patient developed acute pancreatitis as an adverse event.

Recently, Marx et al. [16] enrolled 7 patients with insulinomas <2 cm treated with EUS-RFA in two tertiary centers. All patients showed an immediate relief from symptomatic hypoglycemia after one single session of RFA treatment; 6 of them obtained complete radiological response with a follow-up of 21 months. Adverse effects were reported in two patients (a large retrogastric collection 15 days after treatment and one death one month after the treatment). Strengths of the study include the availability of a structured clinical and radiological FU and the systematic administration of rectal NSAID and antibiotic for, respectively, acute pancreatitis and infection prophylaxis.

Finally, Rossi et al. [17] reported 3 cases of unresectable insulinoma treated with EUS-RFA. A rapid improvement of hypoglycemia symptoms was recorded in 2 patients after the treatment. One patient required a second treatment procedure. In the 24-month follow-up, patients remained asymptomatic and did not show radiological recurrence of the disease.

In summary, most studies evaluating efficacy of EUS-RFA in insulinomas demonstrate rapid symptoms control in virtually all cases with limited adverse events occurrence [14,18].

In these studies, the included F-PanNENs were mostly single lesions with a diameter inferior to 20 mm.

On the other hand, size above 2 cm and higher Ki67 proliferative activity (G2 or G3) are factors usually associated with an aggressive course of the disease, and in these patients the role of EUS-RFA is probably limited [8,9].

Another limitation of the aforementioned studies is the lack of direct comparison with surgery, which at the moment represents the standard of care for these patients.

However, Crinò et al. [19], using propensity score matching, compared safety and efficacy of EUS-RFA (89 pts) and surgical resection (89 pts) of pancreatic insulinomas. Clinical efficacy was 100% after surgery and 95.5% after EUS-RFA (*p* = 0.160), considering that 15 lesions (16.9%) recurred after this treatment. Nonetheless, the adverse event (AE) rate was significantly higher in the surgical group (61.8% vs. 18.0%), and the hospital stay was significantly longer in the surgical group. Additional strengths of this study include the multicenter nature, the high number of patients included and the use of propensity matching in order to limit the bias of the retrospective nature.

If these data are confirmed in the upcoming prospective multicenter randomized trial, EUS-RFA might become the first-line treatment of most patients with insulinomas [20].

Regarding NF-PNENS, Choi et al. [21] collected 7 patients (median diameter of 20.3 mm, range 8–28 mm) who underwent 13 sessions of EUS-RFA. Five patients (71%) achieved a complete response; mild adverse effects (abdominal pain, mild acute pancreatitis) occurred in 2 patients.

Barthet et al. [22] enrolled 12 patients with 14 small non-functioning NF-PNENS (mean diameter 13.1 mm, range 10–20 mm) treated with 50 W ablative power. In 12 patients (86%), a tumor regression was recorded at the 12-month follow-up, and 9 of them obtained complete necrosis after 6 months. Relevant adverse effects included one case of main pancreatic duct stenosis in a patient with treated tumor less than 1 mm distance from the pancreatic duct and a first single case of mild acute pancreatitis, after which prophylactic rectal FANS administration was introduced in the study protocol.

Olienikov et al. [14] evaluated 11 patients with 18 NF-PNENS (mean diameter 17.7 mm) who underwent short cycles (5–12 s) of EUS-RFA, with ablative power of 10–50 W. Follow-up was available for only 9 patients; a single patient showed no complete response to treatment (defined as no change in tumor size or less than 50% size reduction). Among the adverse effects, two cases of mild acute pancreatitis treated conservatively were recorded.

De Nucci et al. [18] reported EUS-RFA treatment with Taewoong’s devices in 10 patients with 11 P-NETs (mean diameter 14.5 mm), including 6 NF-pNEN- and 5 F-pNEN-type lesions. Complete ablation of all 6 NF-pNENs was achieved in a single endoscopic session and confirmed with a CT scan after 6 and 12 months of follow-up. Two cases of abdominal pain were reported as adverse effects.

The retrospective study by Choi et al. [23] reported EUS-RFA treatment of 13 NF-pNENs and 1 F-pNEN (insulinoma), with an average diameter of 12 mm Nine out of thirteen (69%) NF-pNENs treated with Taewoong’s devices and application of ablative power of 50 W obtained a complete response. Two cases of acute pancreatitis were recorded.

A prospective, single-center study by Younis Fadi [24] reported 22 patients with premalignant pancreatic cystic neoplasm and NENs (median size 8.9 mm, range 6–18 mm—6 NF-pNENs), treated with EUS-RFA. A complete radiological response was demonstrated in 4/6 patients. As adverse effects, 2 cases of abdominal pain and 1 case of acute pancreatitis were reported.

In a recent systematic review [25], 61 patients with 73 PNETs (mean diameter of 16 mm, range 4.5–40 mm, F-NETs in 30% of cases) were included from 12 studies. All the patients were treated with EUS-RFA: the effectiveness of the treatment was very high (96%), without significant difference between F-PNETs and NF-PNETs. Nonetheless, the technique demonstrates a high safety profile based on the low rate of adverse events (13.7%), which included 5 instances of post-procedural abdominal pain, 4 mild acute pancreatitis, 1 self-limited fever and 1 necrotizing pancreatitis.

Recently, a large retrospective study conducted by Napoleon et al. [26] focused on the safety and potential predictors of adverse events occurrence after EUS-RFA. A total of 64 NENs (48 NF-PNENs and 16 F-PNENs) were included. Overall, 21 adverse events occurred (7 epigastric pain, 11 acute pancreatitis, 3 main pancreatic duct leak): the proximity (<1 mm of distance) of the pancreatic neoplasm to the main pancreatic duct was the only significant risk factor for AEs occurrence. A similar finding was obtained by another study in which a case of pancreatic duct stenoses occurred after EUS-RFA for a lesion distant less than 1 mm from the pancreatic duct [22]. Together these findings suggest that a distance >1 mm from the main pancreatic duct or the use of prophylactic pancreatic stenting might be warranted in order to limit the occurrence of AE after EUS-RFA.

In summary, EUS-RFA represents an effective treatment also for NF-PNENs, with a high safety profile.

Optimal patient selection for this treatment still remains an open question to be addressed.

### 2.3. Radiofrequency Ablation in Pancreatic Cancer

Almost 90% of pancreatic malignancies are ductal adenocarcinoma (PDAC), which are characterized by a poor prognosis at 1 year (1-year survival rate of 18%) [27]. Different treatments are available according to the clinical stage: from surgical therapy for resectable or potentially resectable disease with the possible addition of adjuvant and neoadjuvant therapy to systemic therapies for unresectable disease. To date, the use of EUS-RFA in PDAC has a cross-sectional role; it can be used at different cancer stages as a multimodal and multidisciplinary treatment approach. For example, it plays a role in the tumor downstaging/debulking [28].

Song et al. [29] evaluated the feasibility and safety of EUS-RFA in 6 patients with unresectable PDAC (locally advanced or metastatic). Mild adverse effects were recorded in 2 patients with mild post-procedural abdominal pain, managed with analgesic therapy.

In following studies, radiological response was also evaluated as an additional endpoint, in patients affected by unresectable pancreatic cancer who were treated with EUS-RFA.

In the study by Scopellitti et al. [30], 10 patients with unresectable locally advanced PDAC after neoadjuvant chemotherapy underwent EUS-RFA with tumor size reduction in half of the patients at 1-month follow-up and without significant side effects.

Similar results were subsequently obtained by Crinò et al. [31] and Wang et al. [32]. A reduction in tumor diameter after treatment with EUS-RFA was reported in both studies: 30% in 7 patients with unresectable locally advanced PDAC enrolled in Crinò’s study and 20% in 11 cases evaluated by Wang.

A pivotal prospective study published in 2022 [33] evaluated the feasibility, safety and efficacy in patients with unresectable PDAC (either locally advanced or metastatic) who underwent EUS-RFA.

In this study, 10 patients were enrolled and received standard chemotherapy treatment in association with EUS-RFA with an average of 1–4 RFA sessions for each patient (total of 22 RFA sessions). The mean diameter of PDACs was 39.2 mm. A 30-month follow-up was performed. Control abdominal imaging, achieved for 9 of the 10 patients, revealed tumor regression in 7 patients (with tumor size reduction greater than 50% in 3 patients), while cancer progression was observed in the remaining 2 patients. The median overall survival was 20.5 months. Particularly, in one case, tumor regression led to the possibility of undergoing standard surgical treatment. No major adverse events occurred after EUS-RFA (follow-up until 4 weeks). Post-procedure abdominal pain was observed in 12 of 22 cases, managed with medical therapy without any hospitalization.

All these studies provided results on the feasibility and safety of the treatment in patients with unresectable PDAC, but unfortunately, important oncological outcomes such as overall survival (OS) and progression-free survival (PFS) were not evaluated.

An exception is represented by the study of Oh and colleagues [34], who enrolled 22 patients with locally advanced (LA) or metastatic (MTX) PDAC treated with EUS-RFA in association with systemic chemotherapy. EUS-RFA was technically feasible in all patients. The median OS and PFS were 24.03 months and 16.37 months, respectively, over a median follow-up period of 21 months. Adverse events occurred in 4/107 (3.74%) RFA sessions and included peritonitis (1) and abdominal pain (3).

In summary, despite the abovementioned interesting results, well-designed comparative studies are needed to better assess efficacy and safety of locoregional ablation in selected patients with PDAC.

### 2.4. Radiofrequency Ablation in Pancreatic Metastases

Pancreatic metastasis from renal cell carcinoma (RCC) occurs in up to 6% of patients after a mean 9.2 years. In these cases, considering the high morbidity rate of surgery, chemotherapeutic and locoregional treatments such as EUS-RFA are often considered.

A recent prospective French study evaluated the effectiveness of EUS-RFA in the largest number of patients with metastatic RCC reported so far [35]. Twelve patients were included and underwent a total of 26 procedures. Control rates at 6- and 12-month follow-up were 84% and 73%, respectively. Adverse events included a case of duodenal abscess and a case of hepatic abscess, which required hospitalization and intervention. The authors concluded that this technique could be an effective treatment to obtain disease control in patients with metastatic RCC.

In summary, in this review, we analyzed 18 studies about EUS-guided RFA for the treatment of solid pancreatic neoplasms, including a total of 311 patients, 232 with pNEN (functional and non-functional), 67 with PDAC and 12 with pancreatic metastases.

The most frequent reported adverse events were abdominal pain (in 33 cases—about 10.6%) and acute pancreatitis (occurred in 18 cases—about 5.8%).

### 2.5. Cryothermal Ablation

Cryothermal ablation is performed using a cryotherm probe (CTP) and combining bipolar RFA with a cooling system based on a cryogenic gas (ERBE Elektromedizin GmbH, Tübingen, Germany). This system is able to overcome some of the limitations of the traditional monopolar RFA.

Recently, Testoni and colleagues performed a randomized controlled trial evaluating the role of EUS cryothermal ablation using a hybrid thermal probe (EUS-HTP) [36]. Patients with LA or borderline resectable (BR) PDAC were randomly allocated in a 1:1 fashion to standard-of-care chemotherapy (CHT) alone or in combination with EUS-HTP. Enrolment was prematurely stopped in 2020 due to slow enrolment rate and device withdrawal from the manufacturer. A total of 17 patients in the combination arm and 20 patients in the CHT alone arm were analyzed. No differences were observed between the two groups in terms of 6-month progression-free survival (PFS), median PFS time, tumor reduction volume and surgical outcomes.

Adverse events occurred in 29.7% of patients and included bleeding at the needle puncture site, jaundice requiring endoscopic biliary stenting, fever, splenic vein thrombosis, asymptomatic perigastric collection and duodenal ulcer at the needle puncture site (resolved with medical therapy).

### 2.6. Photodynamic Therapy

Photodynamic therapy (PDT) utilizes a small-diameter quartz optical fiber to illuminate tissue with laser light through a previously administered photosensitizing agent (such as porfimer sodium). This interaction produces oxygen free radicals, leading to tissue necrosis for death of the tumor cells. The laser fiber can be passed through a standard 19-gauge needle and used under EUS guidance.

DeWitt and colleagues [37] included in a prospective single-center study 12 patients with treatment-naive locally advanced PDACs who underwent EUS-PDT using porfimer sodium as photosensitizer. After 18 days from EUS-PDT, a CT scan was performed to assess the pancreatic necrosis, and chemotherapy treatment was started. In comparison to baseline, the CT scan showed an increase in the percentage of tumor necrosis in 50% of patients. Median follow-up was 10.5 months, median PFS and OS were 2.6 months and 11.5 months, respectively. There were 4 adverse events related to porfimer sodium (sunburned hands, nausea, photosensitivity and skin hyperpigmentation).

In 2021, Hanada et al. [38] evaluated the feasibility of EUS-PDT in 8 patients with LAPD using verteporfin as photosensitizer. After 48 h from treatment, pancreatic necrosis was observed in 5/8 patients. After 14 days, 2 patients reported abdominal pain and one patient accessed an emergency department for hematochezia (probably not related to the treatment).

**Table 1:** Studies about EUS-guided thermal ablative techniques for solid pancreatic neoplasms.

Author, Year	Type of Study	Diagnosis	No. of Patients	Lesion Size (mm)	Type of Therapy	Efficacy	Adverse Events (*n*)	Reference
Choi 2018	Prospective	NF-pNEN F-pNEN	7 1	20.3 12	RFA	5/7 71.4% * 100%	Abdominal pain (1), acute pancreatitis (1) 0	[21]
Barthet 2019	Prospective	NF-pNEN	12	13.1	RFA	12/14 * 86%	Acute pancreatitis (1), MPD stenosis (1), fever (1), extrapancreatic necrosis (1)	[22]
Oleinikov 2019	Retrospective	NF-pNEN F-pNEN	11 7	17.7 15.3	RFA	15/18 * 83.3% 7/7 100%	Acute pancreatitis (2) 0	[14]
Younis 2019	Case series	NF-pNEN	3	10	RFA	Not reported	Abdominal pain (1)	[24]
De Nucci 2020	Case series	F-pNEN NF-pNEN	5 5	12.8 16	RFA	5/5 * 6/6	0 Abdominal pain (2)	[18]
Furnica 2020	Case series	F-pNEN	4	12.9	RFA	4/4 *	Acute pancreatitis (1), abdominal pain (1)	[15]
Choi 2020	Retrospective	F-pNEN NF-pNEN	1 13	12 18.1	RFA	100% * 9/13 69%	0 Acute pancreatitis (2)	[23]
Rossi 2022	Case report	F-pNEN	3	9–14 mm	RFA	100% *	Procedural bleeding (1)	[17]
Crinò 2023	Retrospective	F-pNEN	89	-	RFA	95.5%	No severe adverse events compared to surgical group	[19]
Marx et al., 2022	Retrospective	F-pNEN	7	13.3	RFA	85.7% *	Large retrogastric collection (1), minor adverse events (3)	[16]
Napoleon 2023	Retrospective	NF-pNENs F-pNENs	48 16	15	RFA	33/48 (71.7%) of NF-pNENs complete response and 12 partial response 12/16 complete response (80%) of F-pNENs and 3/16 partial response	Epigastric pain (7), acute pancreatitis (11), main pancreatic ductal leak (3)	[26]
Song 2016	Prospective	LA and MTX PDAC	6	38	RFA	Successfully performed in all 6 patients	Abdominal pain (2)	[29]
Scopelliti 2018	Prospective	Unresectable PDAC	10	49.2	RFA	Tumor size reduction in 50% of patients	Abdominal pain (2)	[30]
Crinò 2018	Prospective	Unresectable PDAC	8	30.6	RFA	Tumor side reduction in 30% of patients	Mild abdominal pain (3)	[31]
DeWitt 2018	Prospective	LA PDAC	12	45.2	PDT	Increase in tumor necrosis in 50% of patients at CT scan control	4/12 (related to porfimer sodium), including sunburned hands, nausea, photosensitivity and skin hyperpigmentation)	[37]
Testoni 2021	Randomized clinical trial	LA and BRSEC PDAC	17	33.3	Two arms (CTP + CHT vs. CHT)	No differences in tumor reduction volume	11/37 sessions (29.7% of patients (including fever, jaundice, perigastric collection, splenic vein thrombosis)	[36]
Wang 2021	Retrospective	Unresectable PDAC	11	28	RFA	Tumor size reduction in 20% of patients	0	[32]
Chanez 2021	Prospective	Pancreatic metastases	12	17	RFA	Complete radiological response in 40% at 12 months. Control rate 73.3% at 12 months	2/12 duodenal abscess (1) and hepatic abscess (1)	[35]
Thosani 2022	Prospective	Unresectable PDAC	10	39.2	RFA	Tumor regression in 7 patients	Mild abdominal pain (12) in 22 RFA sessions	[33]
Oh 2022	Prospective	LA and MTX PDAC	22	38	RFA	Successfully performed in all patients	4/107 sessions, including peritonitis (1) and abdominal pain (3)	[34]
Hanada 2022	Prospective	Unresectable PDAC	8	33.3	PDT	5 lesions with necrosis at CT control	0	[38]

* Efficacy defined as disappearance of symptoms for F-pNEN and disappearance of the lesion at cross-sectional imaging during follow-up for NF-pNEN. CTP, Cryothermal ablation; PDT, photodynamic therapy; RFA, radiofrequency ablation; LA, locally advanced; MTX, metastatic; BRESEC, borderline resectable; PDAC, pancreatic adenocarcinoma, CT, computed tomography, pNENs, pancreatic neuroendocrine neoplasms; NF, non-functional; F, functional.

## 3. Non-Thermal Injection Techniques

Several EUS-guided fine-needle injection (FNI) therapies have been evaluated in efficacy and feasibility studies. The most commonly studied non-thermal injection technique is represented by ethanol injection (EI). In addition to EI, other EUS-guided injection therapies included immunotherapy, the application of viral vectors, locoregional chemotherapy, brachytherapy and gene transfer therapies.

Studies about non-thermal injection procedures are summarized in Table 2.

### EUS-Guided Ethanol Injection

Ethanol injection is performed with a common EUS-FNA needle and does not require any specific device. The amount of injected alcohol varies between the available studies and ranges from 0.3 to 8.0 mL per session, with a concentration varying from 50% to 99%, with the latter being the most commonly used. The mechanisms through ethanol act include cellular dehydration, proteins denaturation and vasculature occlusion with the final induction of coagulative necrosis [39].

EI has been performed mostly for the treatment of PNENs and pancreatic cystic lesions, while studies on PDAC are limited. The effectiveness of EUS-EI in pNENs has been evaluated in multiple case reports and small case series, while prospective multicenter trials are not available.

The first studies in this field focused on patients with insulinomas at high surgical risk and, subsequently, also on NF-PNENs.

The first case of successful EUS-EI for insulinoma was reported by Deprez in 2008 [40].

**Table 2:** Studies about EUS-guided non-thermal injection techniques for solid pancreatic neoplasms.

Author, Year	Study Type	Disease	No. of Patients and Groups	EUS-Guided Injectable Agents	Type of Therapy	Clinical Success	Adverse Reactions (AEs)	Reference
Deprez 2008	Case report	F-pNEN	1	Ethanol volume 3.5 mL	Ethanol injection	100%	Duodena hematoma and bleeding (1)	[40]
Levy 2012	Case series	F-pNEN	5	Ethanol volume 0.8 mL	Ethanol injection	5/5 (100%)	None	[41]
Choi 2018	Prospective	NF-pNENs F-pNEN	32 1	Ethanol volume 1.1 mL	Ethanol injection	24/40 (60%)	Acute pancreatitis (2) with PD stricture (1)	[42]
Choi 2023	Retrospective	NF-pNENs F-pNENs	40 7	Ethanol dose > 0.35 mL/cm^3^	Ethanol injection	8 cases of complete response	Acute pancreatitis (11), pancreatic enzyme elevation (4), duodenal stricture (1).	[43]
Chang 2000	Phase I	Unresectable PDAC	8, single arm	Allogeneic mixed lymphocyte culture	Immunotherapy	Partial remission 25%, minor response 12.5%	Dose-limiting toxicity (DLT) 0	[44]
Irisawa 2007	Pilot clinical study	Unresectable PDAC refractory to gemcitabine	7, single arm	Dendritic cells (DCs)	Immunotherapy	Mixed response 28.6%, stable disease 28.6%	None	[45]
Hirooka et al., 2009	Phase I	Locally advanced pancreatic cancer (LAPC)	5, single arm	OK-432-pulsed DCs	Immunotherapy	Effective response 60% (partial remission 20%, stable disease 40%)	4 patients with Grade 3 AEs, 1 patient with Grade 1 AEs	[46]
Hirooka et al., 2017	Phase I/II	LAPC	15, single arm	Zoledronate-pulsed DCs	Immunotherapy	Stable disease 46.7%	DLT 0 (grade 3 AEs: 4)	[47]
Levy et al., 2017	Prospective, not randomized	Unresectable PDAC	36, single arm	Gemcitabine	Chemotherapy	Partial response 25%, stable disease 57%	None	[48]
Hanna et al., 2012	Phase I/II	Unresectable PDAC	9, two cohorts	BC-819 DNA plasmid	Genic Therapy	Partial response in 3 patients	Asymptomatic elevation of lipase (1)	[49]
Buscail et al., 2015	Phase I	LAPC	22	Complexed plasmid/CYL-02 + gemcitabine	Genic Therapy	Stable disease in 12 patients	None	[50]
Golan et al., 2015	Phase I/II	LAPC	15	Intratumoral placement of SiG12-LODER *+* gemcitabine	Genic therapy	Partial response in 2 patients; stable disease in 10 patients	Mild side effects (Grades 1 and 2) in 90% of cases	[51]
Hecht et al., 2003	Phase I/II	Unresectable without liver metastasis	21, single arm	ONYX-015	Viral therapy	Partial response 10%, stable disease 38%	8 patients with AEs (4 due to viral therapy, 4 due to injection technique)	[52]
Hecht et al., 2012	Phase I/II	LAPC	50, single arm	TNFerade biologic	Viral therapy	Complete response 2%, partial response 6%, minor response 8%, stable disease 24%	DLT 3	[53]
Herman et al., 2013	Randomized Phase III	LAPC	304, two arms	TNFerade biologic	Viral therapy	No difference	No difference in Grade 3 or 4 AEs	[54]
Sun et al., 2006	Pilot trial	Unresectable PDAC	15, single arm	Iodine-125	Brachytherapy	Partial response 27%, minimal response 20%, and disease stabilization 33%	AEs 6	[55]
Jin et al., 2008	Prospective pilot study	Unresectable PDAC	22, single arm	Iodine-125	Brachytherapy	3 cases of partial remission, 10 cases of stable disease	None	[56]
Bhutani et al., 2019	Case report	Unresectable PDAC	1	Phosphorous-32 (32P) microparticles	Brachytherapy	Reduction of 58% of tumor volume at week 16	None	[57]
Naidu 2021	Pilot study	LPAC	12, single arm	Phosphorous-32 (32P) microparticles	Brachytherapy	Median reduction in tumor volume was 8.2 cm3, tumor downstaging in 6 patients, resection in 5 (R0 in 4 patients).	None	[58]
Ross et al., 2022	Pilot study	Unresectable LPAC	42, single arm	Phosphorous-32 (32P) microparticles	Brachytherapy	Local disease control rate was 97.3%	AEs 41 in 16 patients treated (including 8 grade 3 AEs in 3 patients)	[59]

Subsequently, Levy and colleagues described a case series [38] of 5 patients with insulinomas (mean diameter 17 mm) treated with 11 sessions of EI (0.8 mL mean ethanol volume per session) and demonstrated an efficacy of 100% without relevant side effects.

Choi et al. retrospectively evaluated the efficacy of EI in patients with small PNENs [42]. A total of 33 patients with 40 PNENs (39 with NF-NENs and 1 with insulinoma—diameter less than 2 cm) were included in the study. A total of 63 EUS-EI sessions were performed (1.6 sessions per patient) with a median volume of ethanol injected of 1.1 mL. Complete tumor ablation, defined as the complete absence of enhancement at imaging follow-up, was demonstrated in 24/40 cases (60%). Acute pancreatitis occurred in 2 patients (3.2%), and it was the only AE observed.

More recently, the same group performed a similar study evaluating potential predictive factors of response to EUS-EI [43]. A total of 72 patients (40 NF-PNENs, 7 F-PNENs and 25 solid pseudopapillary tumors) were included retrospectively. At multivariate analysis, ethanol dose (>0.35 mL/cm^3^) and the histological diagnosis of PNENs appeared to affect treatment response.

Finally, a recent systematic review and meta-analysis performed by Garg and colleagues evaluated the efficacy and safety of EUS-EI in pNENs [60]. Overall, 91 EUS-EI in 81 patients were performed. Technical and clinical success rates were 96.7% and 82.2%, respectively. The AEs rate was 11.5%, with acute pancreatitis representing the most common AE (7.6%).

## 4. Other Non-Thermal Injection Techniques

The injection of other substances, besides ethanol, under EUS guidance have been evaluated in small feasibility studies and mostly in patients with PDAC.

The basic concept of these therapies is to inject various antitumoral agents directly into the target lesion in order to enhance their intratumoral concentration and to reduce systemic side effects.

The precise role of injection techniques in PDAC has not yet been defined; however, reduction of the tumor mass/debulking and boosting of chemotherapies’ efficacy might be expected.

### 4.1. Immunotherapy

The rationale of immunotherapy is to block the tumor-produced inhibitors of the immune system and to reactivate the immune system against the cancer cells. This mechanism could enhance the available strategies for cancer treatment and promote tumor regression.

Studies about EUS-guided FNI immunotherapy in PDACs have been conducted with allogeneic lymphocyte cultures and with dendritic cells.

Chang et al. performed the milestone study about this new technique in patients with PDAC [44]. This phase 1 trial included 8 patients affected by unresectable PDAC treated with intratumoral injections of allogenic mixed lymphocyte cultures (from donors and from patients). Escalating doses of 3, 6 and 9 billion implanted cells were injected via EUS-guided FNI in a single session. Two partial efficacy responses and one minor response were obtained. Median survival was 13.2 months. Minor side effects, such as nausea and fever, were reported and managed conservatively.

The only prospective study about locoregional chemotherapy (gemcitabine) [48] enrolled 13 patients with MTX PDAC and 22 patients with LA PDAC. All patients underwent EUS-guided FNI of gemcitabine. Patients were also sequentially treated with standard multimodal therapy, such as chemotherapy alone and chemoradiation therapy. No adverse effects were observed. Among 20 patients with unresectable and LA disease (stage III), 4 patients achieved downstaging of the disease and could subsequently be resected.

The tumor injection of immature dendritic cells (DCs) represents another locoregional immunotherapy for the treatment of PDAC. Dendritic cells are used as tumor antigen-presenting cells in order to activate the host T-cell immune response against the tumor at the regional nodes.

Several clinical studies have been reported in the literature, including the first by Irisawa [45] and two by Hirooka [46,47]. In these studies, the efficacy and safety of this technique were demonstrated, even if new studies will be needed to evaluate the best strategy to apply DCs and the best associated therapeutic combination.

Locoregional therapies with oncolytic viruses (OVs) should also be mentioned. These therapies exploit the advantages from the oncolytic and replicative power of specific viruses, which may be engineered. They allow the expression of target genes within the tumor site, where they may disseminate and replicate, in order to provoke cytotoxic effects against tumor cells. Experiences reported in the literature involve both TNFerade and ONYX 015.

TNFerade is an adenovirus-deficient vector that carries the gene directly into the tumor for the production of tumor necrosis factor (TNF) alpha, a cytokine with anticancer activity [54] that is produced by the inflammatory system. Hecht et al. conducted a phase I/II study including 50 patients with LA PDAC who were treated with 5 weekly EUS-guided injections of TNFerade, in addition to chemotherapy and 5-fluorouracil radiotherapy. The procedure was well tolerated; however, a clinical response to treatment was observed in a few patients [53]. A randomized phase 3 trial with TNFerade combined with fluorouracil and radiotherapy in LA PDAC was conducted by Herman et al. [54]. In this study, the use of TNFerade in 304 patients combined with standard treatment was safe, but it did not show efficacy in terms of prolonged survival.

A phase I/II study by Hecht et al. [52] used EUS-guided injection of ONYX-015 in 21 patients with advanced PDAC. ONYX-015 is a modified adenovirus that is able to target cancer cells to reproduce itself inside the cancer cells inducing their death. In this study, 21 patients were treated in combination with standard chemotherapy (gemcitabine), but only 2 patients showed partial tumor regression, while 11 patients showed a progression of the disease. Two cases of duodenal perforation occurred, ascribed to the rigidity of the endoscope.

### 4.2. EUS-Guided Radiotherapy

Stereotactic body radiation therapy (SBRT) is a nuclear medicine technique characterized by the application of high radiation doses into a specific cancer site by a cyberknife or similar. This technique needs fiducial markers placement to maximize the targeting accuracy. Traditionally, CT scan and percutaneous ultrasound imaging have been the conventional guidance to position the fiducial markers in PDACs. However, these approaches present some limitations, as the pancreas is a retroperitoneal organ, and percutaneous insertion could increase the risk of complications, such as vascular injury, reduced therapeutic efficacy or peritoneal dissemination of the cancer.

In 2014, Choi et al. evaluated the safety and feasibility of EUS fiducial placement for SBRT [61], showing a technical success rate of 100% (32/32) and lower risk of peritoneal tumor dissemination during EUS-guided fiducial placement compared to percutaneous approaches.

Park et al. [62] employed EUS guidance as an insertion marker for SBRT in 57 patients with unresectable PDAC and demonstrated a reduced risk of hemorrhage with real-time Doppler function (only minor hemorrhage was reported in one patient).

### 4.3. Brachytherapy

Brachytherapy consists in the insertion of radioactive substances into the cancer site. In this setting, EUS could play a crucial role as a guide for the injection of radioactive sources. Iodine-125 (which has the longest half-life and is more effective for rapid-growth PDACs) and phosphorus-32 are the most widely used radioactive substances. At present, no approved brachytherapy technique has been established for the treatment of PDAC. The following studies about EUS-guided brachytherapy are overviewed in the literature; the major trials used iodine-125 and new approaches with phosphorus-32.

Sun et al. [55] reported a study in which 15 patients with unresectable PDAC (either locally advanced or metastatic) were implanted with an average of 22 radioactive iodine-125 seeds, delivered under EUS guidance into the tumor. The mean follow-up was 10 months. Endpoints included performance status evaluation (according to Karnofsky) and pain response, tumor response (assessed by CT and/or EUS) and survival. Median survival was 10.6 months. At follow-up, partial response was observed in 27% of patients, minimal response in 20% of patients and disease stabilization in 33% of patients. Thirty percent of patients showed pain reduction. As adverse effects, acute pancreatitis and pseudocysts occurred in 3 patients, whereas 3 patients suffered from hematologic toxicity (anemia, neutropenia, thrombocytopenia), without severe clinical sequelae.

In 2008, Jin. Z et al. reported a study in which 22 patients with advanced PDAC underwent EUS-guided radioactive iodine seeds insertion [56]. All patients were successfully implanted with iodine seeds via EUS and received standard chemotherapy (gemcitabine-5 fluorouracil) a week after brachytherapy. During the 9 months of follow-up, the estimated median survival time was 9 months. A pain reduction was recorded one week after brachytherapy, but it worsened after one month. No complications occurred after brachytherapy.

Finally, the use of EUS-guided phosphorus-32 injection for the treatment of PDAC appears to be a novel promising approach. In 2019, Bhutani et al. [57] first reported a case of a 72-year-old patient with LA PDAC treated with standard chemotherapy (gemcitabine + nab-paclitaxel) together with EUS-guided phosphorus-32 injection. A positron emission computed tomography (SPECT-CT) was performed 4 h and 7 days after radioactive seed insertion to check the appropriate distribution of radioactive particles into the cancer site. CT scans were performed every 8 weeks to assess cancer response, and the reduction of 58% of tumor volume was observed at week 16. A reduction of carcinoembryonic antigen 19.9 (Ca 19.9) and a complete remission of abdominal pain were reported. No side effects occurred, and the patient continued the chemotherapy schedule.

Subsequently, Naidu and colleagues [58] performed a pilot study in 12 patients with LA PDAC treated with a combination of standard chemotherapy and EUS-guided phosphorus-32 injection. A technical success of 100% was reported. A reduction in tumor volume was reported after 12 weeks (median reduction of 8.2 cm3), and it was associated with cancer downstaging, allowing curative surgery in 5 patients. There were no adverse events related to the phosphorous-32 injection reported.

Ross and colleagues recently reported the results of an international multicenter open-label pilot study (PanCO trial) on the use of EUS-guided phosphorus-32 injection in patients with unresectable PDAC associated with standard-of-care chemotherapy [59]. EUS-guided phosphorus-32 injection demonstrated a good safety profile (41 treatment-emergent adverse events (TEAEs) in 16 patients) with also a clinically relevant benefit from the combination therapy (82% local disease control rate at 16 weeks in the intention-to-treat (ITT) analysis and 90.5% in the per-protocol (PP) analysis).

Finally, a prospective, multicenter, randomized trial (TRIPP-FFX trial) comparing standard chemotherapy alone or associated with phosphorus-32 brachytherapy in patients with unresectable locally advanced PDAC is currently in progress (NCT05466799).

### 4.4. EUS-Guided Genic Therapy

Recently, genic therapy gained a prominent role as a targeted therapy for the treatment of pancreatic cancer. There are different genic therapy techniques that may be employed, ranging from the reintroduction of a missing or unexpressed gene, to selective modulation of specific genes, to inhibition of the expression of a specific gene (such as an oncogene) [63].

Three important studies about EUS-guided genic therapies should be mentioned.

In 2012, Hanna et al. [49] published a study involving BC-819 as a gene therapy. BC-819 is a double-stranded DNA plasmid developed to target diphtheria toxin A (DTA) gene expression under regulatory sequences H19 control. H19 is a paternally imprinted oncofetal gene that encodes an RNA without a protein product, which acts as a riboregulator. H19 is upregulated in cancer cells, and its expression is associated with higher risk of early tumor recurrence. The DTA expression results in selective cancer cells destruction via inhibition of protein synthesis, allowing for targeted cancer treatment [64]. BC-819 could potentially treat pancreatic cancer in patients with H19 gene tissue overexpression.

The aim of the study conducted by Hanna et al. [49] was to evaluate safety, tolerability and preliminary efficacy of BC-819 administered inside the cancer in patients with unresectable, locally advanced, non-metastatic pancreatic cancer. Nine patients with unresectable pancreatic adenocarcinoma were enrolled and divided into two cohorts, with escalating doses of BC-819 administered twice weekly for 2 weeks. All patients received BC-819 infusion; six patients by EUS-guided technique and three patients by CT-guided approach. At week 4, a PET-CT scan showed no increase in tumor size. Two patients who received additional chemotherapy and chemo-radiation therapy showed downstaging and were considered surgically resectable. There was only one asymptomatic case of increased serum lipase.

In 2015, Buscail et al. [50] conducted a phase 1 French study with the aim to explore the safety and preliminary clinical activity of CYL-02. CYL-02 is a nonviral genic therapy product that improves chemotherapy susceptibility of pancreatic cancer cells. Twenty-two patients suffering from pancreatic cancer treated with gemcitabine active chemotherapy were included in the study. Nine patients showed cancer stability at 6-month follow-up. No serious side effects were reported.

Among genic therapy techniques, the intratumoral RNA interference (RNAi) use in patients with KRAS-mutated pancreatic cancer expressing should be mentioned. Golan et al. [51] applied a particular type of RNAi against KRAS for the treatment of LAPCs (siG12D-LODER), which is a biodegradable polymeric matrix that allows a prolonged release of RNAi inside the cancer during several months.

In this phase 1/2 study, 15 patients with LAPC, treated with gemcitabine, were enrolled and treated with a single intratumoral injection of EUS/CT-guided siG12D-LODER.

CT scan control showed no cancer progression in 12 patients, a disease stability in 10 patients, while a partial response occurred in 2 patients. Mild side effects (grades 1 and 2) were reported in 90% of cases.

## 5. Conclusions

To date, despite the rapid evolution in this field, EUS-guided locoregional treatments for solid pancreatic neoplasms are still performed mostly in the context of clinical trials and research.

So far, EUS-RFA represents the most studied procedure, especially for the treatment of PNENs.

In particular, EUS-RFA might soon become the standard of care for the treatment of F-PNENs, as available data in these patients already demonstrated very high rates of efficacy and low adverse events occurrence.

The impact of other locoregional treatments, especially in patients with PDAC, needs to be carefully investigated in properly designed study, preferably randomized as the available data are still very limited.

Finally, numerous other neoplasms or metastatic lesions might benefit from EUS-guided locoregional treatment, potentially opening new scenarios in the treatment algorithm of various neoplastic conditions.

## Data Availability

No new data were created or analyzed in this study. Data sharing is not applicable to this article.
